# DOES LIVING WITH A FAMILY AFFECT SOCIAL CONTACT/PARTICIPATION? VARIATIONS ACROSS BIRTH COHORTS AND GENDER IN JAPAN

**DOI:** 10.1093/geroni/igad104.1583

**Published:** 2023-12-21

**Authors:** Erika Kobayashi

**Affiliations:** Tokyo Metropolitan Institute for Geriatrics and Gerontology, Tokyo, Tokyo, Japan

## Abstract

Changing family structures and values might have affected social relationships outside the household in a different way for men and women. This study clarified birth cohort and gender differences among older Japanese in terms of (a) trajectories of social contact and participation and (b) effects of living with a family on them. Data were obtained from ten waves of the National Survey of the Japanese Elderly (1987-2021) for 5,855 individuals who commenced participation by 2012. Quadratic growth models were examined as a function of age at time t (≥63 years) for the frequency of contact with children living apart, with friends and neighbors, and participation in community groups, whose intercept and slope were predicted by gender and birth cohorts (C1:1902-13, C2:1914-25, C3:1926-37, C4:1938-49). Whether effects of being married and co-residing with a child would differ by gender and cohorts was also examined, controlling for socioeconomic and health status. The results showed that the level of contact/participation at age 75 increased in women in recent cohorts. The frequency for friends and groups declined with age after the mid-70s across cohorts. C4 showed a steeper decline in the child/friend contact, indicating the COVID-19 influence. Living with a family generally impeded social contact, except that married men contacted non-coresident children more frequently than their unmarried counterparts. The negative effect of living with a child was greater in women and the earliest birth cohort. Thus, social isolation could matter not only for older adults living alone, but also for those living with a family.

